# Impact of the CYFIP2 R87C variant in a human neuronal model in vitro

**DOI:** 10.1038/s41598-026-44176-2

**Published:** 2026-03-18

**Authors:** Isabelle Zaboroski-Silva, Evelin da Silva Brandão, Bianca de Freitas Brenha, Curran Landry, João Carrara, Rodolfo Sanches Ferreira, Isadora May Vaz, Valderez Ravaglio Jamur, Ashleigh E. Schaffer, Helen Cristina Miranda, Patrícia Shigunov

**Affiliations:** 1 Stem Cell Basic Biology Laboratory, Instituto Carlos Chagas, Fiocruz PR, Curitiba, PR 81310-020 Brazil; 2 Department of Genetics and Genome Sciences, Case Western Reserve University, Cleveland, OH 44106-4955 USA; 3 Core for Cell Technology, School of Medicine, Pontifícia Universidade Católica do Paraná, Curitiba, PR 80215-901 Brazil; 4 Department of Molecular and Medical Genetics, Oregon Health and Science University, Portland, OR 97239 USA; 5 Department of Neurosciences, Case Western Reserve University, Cleveland, OH 44106-4955 USA; 6 Center for RNA Science and Therapeutics, Case Western Reserve University, Cleveland, OH 44106-4973 USA

**Keywords:** CYFIP2 variant, Neurodevelopment, Human pluripotent stem cell, Disease modeling, Cell biology, Developmental biology, Neuroscience, Stem cells

## Abstract

Mutations in the CYFIP2 gene, particularly the R87C variant, are associated with severe epileptic encephalopathy, and present challenges for therapeutic development. This study utilized CRISPR/Cas9-edited human pluripotent stem cell (hPSC) lines to investigate the impact of R87C variant on neuronal morphology and function. hPSCs were differentiated into neural progenitor cells (NPCs), cortical neurons (CNs), and cortical organoids. Phenotypic characterization included immunofluorescence, scanning electron microscopy (SEM), high-throughput scanning (HTS), multi-electrode array (MEA) recordings, and Western blotting. Edited hPSC lines maintained pluripotency, and neurogenic differentiation yielded NPCs and CNs without significant differences in neural progenitor marker expression. However, mutated NPCs exhibited reduced motility in cell tracking assays, and SEM revealed altered cell morphology, suggesting an impact on lamellipodia formation. While both mutant and wild-type CNs expressed appropriate neuronal and glial markers and showed similar electrophysiological properties, R87C/R87C cortical organoids displayed decreased CYFIP2 protein levels and, by day 30, showed increased size alongside an absence of SOX2 + cells, suggesting premature depletion of the progenitor pool. These findings highlight a marked divergence between 2D and 3D models, with organoids revealing neurodevelopmental abnormalities not evident in monolayer cultures. Together, our results suggest that the CYFIP2 R87C variant impacts NPC cytoskeletal dynamics and early cortical development, warranting further investigation into its role in epileptic encephalopathy.

**Supplementary Information:**

The online version contains supplementary material available at 10.1038/s41598-026-44176-2.

## Introduction

CYFIP2 is a gene that encodes a protein involved in neurogenesis during central nervous system development^1^. It plays a role in branched actin polymerization as part of the WAVE regulatory complex (WRC)^2–4^. In this complex, CYFIP2 interacts with the VCA domain of WAVE, keeping it inactive^5,6^. When new branched actin filaments need to be polymerized, Rac1, a GTPase, binds to CYFIP2, changing its conformation, and releasing the VCA domain to interact with the Arp2/3 complex, which initiates the polymerization^7^. CYFIP2 also interacts with FMPR, an RNA binding protein, which is involved in the post transcriptional regulation of a set of genes associated with neurogenesis and neurodevelopment^8–10^. The exact role of CYFIP2 in the post transcriptional regulation remains unclear.

In the past few years, CYFIP2 variants have been associated with neurodevelopment diseases, such as early infantile epileptic encephalopathy^11–16^. Patients have intellectual disability, infantile spasms, hypsarrhythmia, and microcephaly^14–16^. Among the CYFIP2 variants associated with the disease, the R87C variant causes the most severe symptoms^15^. Recently, an animal model with the R87C variant was described in the literature, which despite the decrease in the brain size and in the cortical area, the R87C mice did not recapitulate the spontaneous seizures of the patients^17^. In that matter, there is need to establish human models to study the phenotype of the variant.

Patient’s induced pluripotent stem cells (iPSC) are increasingly used for disease modeling and drug screening^18,19^, including personalized medicine in genetic epilepsies, enabling the study of disease mechanisms and the development of more efficacious treatments^20^. Our group recently published the development of a human iPSC model from a patient with epileptic encephalopathy carrying the CYFIP2 R87C variant^21^. Here, we CRISPR engineered the isogenic cell derived from the hiPSC line and generated a hESC homozygous for the R87C variant. We induced these cells to neurogenic differentiation in monolayer and to cortical organoids. We observed that neural progenitor cells with the R87C variant presented a decrease in CYFIP2 expression, reduced lamellipodia formation and cell motility, but the cortical neurons did not present a different morphological or electrophysiological phenotype compared to the wild type cells. Also, the presence of the R87C variant led to absence of progenitor cells on the cortical organoid model. The results described here could help to understand the cellular impact of the CYFIP2 R87C variant during neurogenic development.

## Results

### hESCs carrying the homozygous CYFIP2 R87C variant maintain pluripotency

**Fig. 1:**
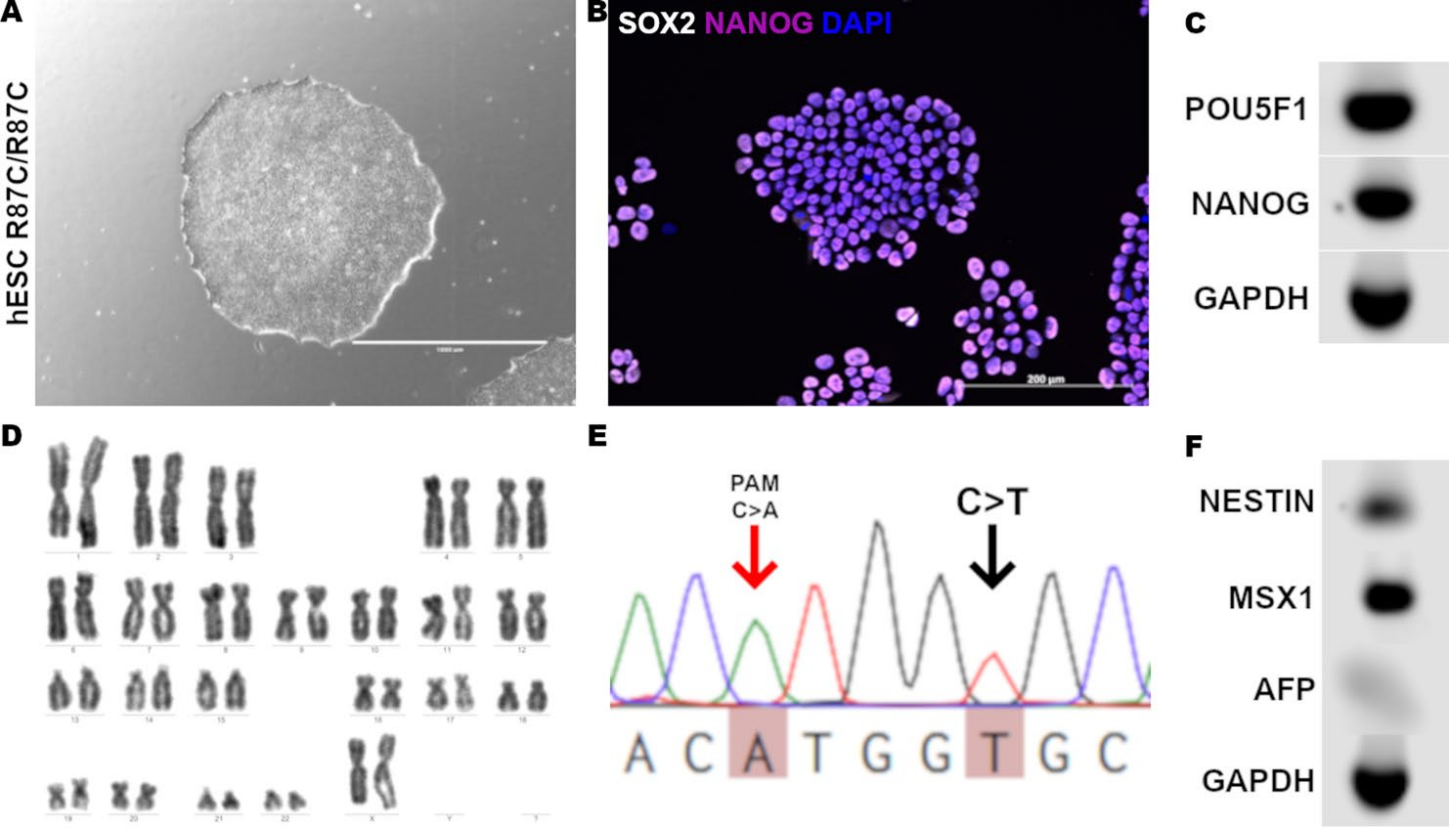
Analysis of hESC after introduction of R87C variant using CRISPR/Cas9 editing. (**A**) Representative image of a hESC colony post-gene editing. Scale bar: 1000 μm. (**B**) Representative immunofluorescence image identifying pluripotency markers in edited hESC. SOX2: white. NANOG: purple. DAPI: blue. Scale bar: 200 μm. (**C**) Gel showing pluripotency markers *POU5F1* (OCT4) and *NANOG*. *GAPDH* served as housekeeping gene. (**D**) Representative G-band karyotype showing no chromosomal abnormalities post-gene editing. (**E**) Electropherogram displaying the C > T mutation on exon 4 of the CYFIP2 gene and the silent C > A mutation in the PAM sequence. (**F**) Gel showing germ layer markers *NESTIN* (ectoderm),*MSX1* (mesoderm), and *AFP* (endoderm) after spontaneous EB differentiation. GAPDH served as housekeeping gene. Full, uncropped gels corresponding to the panels C and F in this figure are shown in Supplementary Fig. 2.

To study the impact of the R87C variant in the *CYFIP2* gene, we introduced the R87C point mutation (c.260G > A) into the H9 hESC line using CRISPR/Cas9 editing (Figure S1). Following clonal selection, the engineered cell lines retained normal karyotypes and expression of pluripotency markers, confirming maintenance of their pluripotent state (Fig. 1A-D, F; Figure S2). Sanger sequencing confirmed the precise genetic modifications (Fig. 1E) and analysis of selected off-targets sites revealed no alterations in their sequences (Figure S3). Together, these results demonstrate that the homozygous *CYFIP2* R87C hESC line preserves pluripotency and is suitable for subsequent neurogenesis analysis.

### The CYFIP2 R87C variant alters motility and gene expression in hESC-derived neuronal progenitor cells

**Fig. 2:**
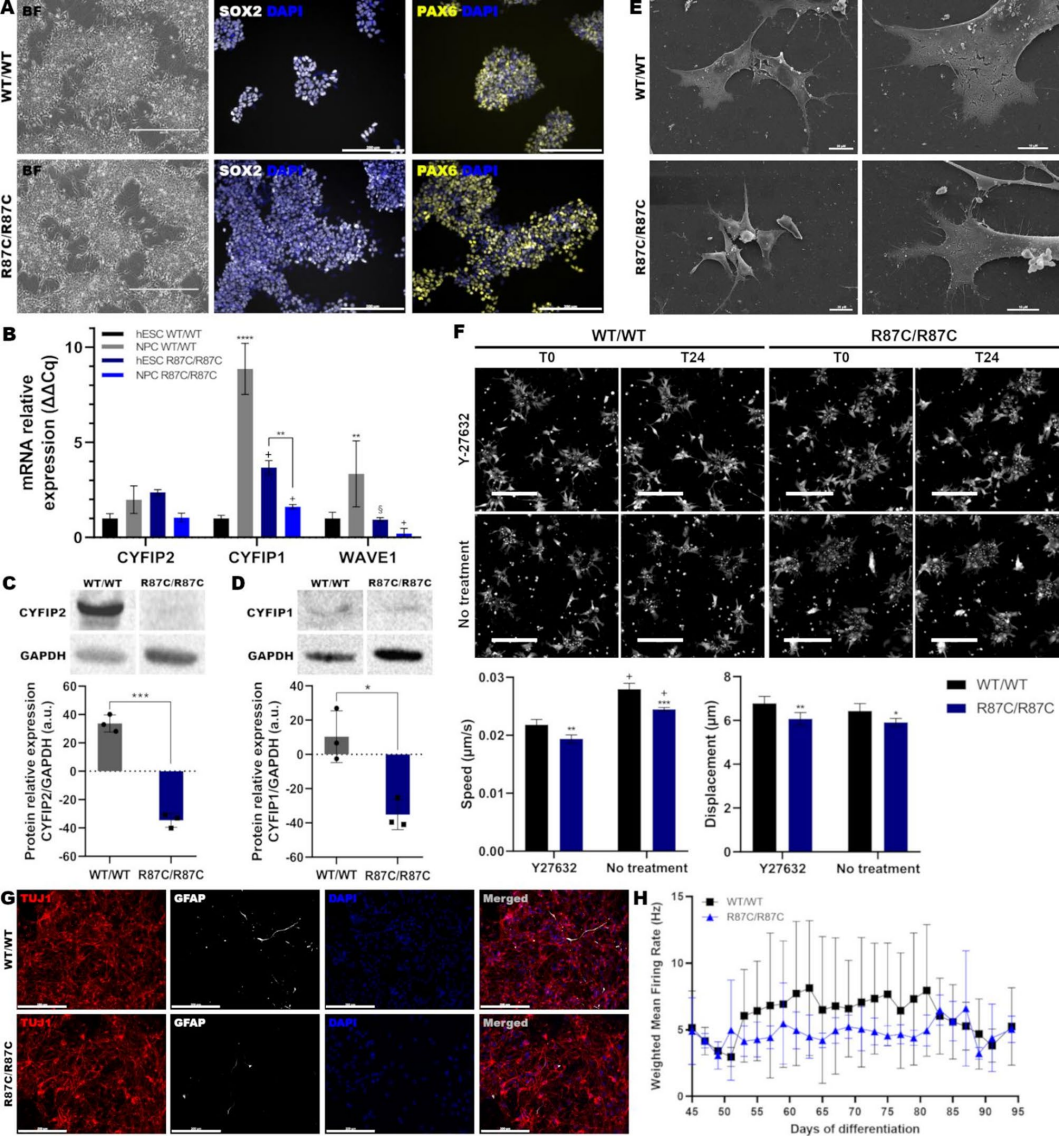
Analysis of R87C/R87C cell line during 2D neurogenic differentiation. (**A**) Representative brightfield images of hESC-derived neural progenitor cells (NPCs), showing expression of NPC markers SOX2 (white) and PAX6 (yellow). DAPI: blue. Scale bar: 200 μm. (**B**) Expression of *CYFIP2*, *CYFIP1*, and *WAVE1* post-NPC differentiation induction. *n* = 3. Statistical analysis: Mean ± SD (hESC WT/WT vs. NPC WT/WT: **p ≤ 0.01, ****p ≤ 0.0001; hESC or NPC R87C/R87C vs. NPC WT/WT: §*p* ≤ 0.01, +*p* ≤ 0.0001). (**C**) Western blot analysis of CYFIP2 and (**D**) CYFIP1 expression in NPCs. GAPDH served as housekeeping protein. *n* = 3. Statistical analysis: Mean ± SD (*p ≤ 0.05, ***p ≤ 0.001). Full, uncropped images of these blots are provided in Supplementary Fig. 4. (**E**) Representative SEM image showing NPC morphology of WT and R87C cells. Scale bars: 100 (left) and 10 (right) µm. (**F**) Cell tracking analysis of NPCs, with or without ROCK inhibitor Y-27632, at timepoint 0 (T0) and timepoint 24 (T24). Plots represent mean speed and displacement over 25 timepoints. Scale bar: 200 μm. Statistical analysis between genotypes (WT/WT vs. R87C/R87C): **p ≤ 0.01, ***p ≤ 0.001. Statistical analysis comparing conditions to WT/WT treated with Y-27632: +*p* ≤ 0.0001. (**G**) Representative immunofluorescence image after 21 days of cortical neuron (CN) differentiation. TUJ1: red. GFAP: white. DAPI: blue. Scale bar: 200 μm. (**H**) Plot representing weighted mean firing rate of CNs during multi-electrode array (MEA) analysis between days 45 and 95 of differentiation. *n* = 3.

To evaluate the impact of the CYFIP2 R87C variant on human neural development in vitro, hESC-derived NPCs were generated and characterized. Both R87C/R87C and wild-type (WT) lines successfully differentiated into NPCs, as indicated by the expression of canonical NPC markers SOX2 and PAX6 (Fig. 2A). Analysis of *CYFIP2*,* CYFIP1*, and *WAVE1* revealed distinct expression patterns. In WT cells, *CYFIP1* mRNA and protein levels were increased at the NPC stage relative to hESC, this increase was absent in R87C/R87C lines (Fig. 2B-D; Figure S4A-B). Importantly, *CYFIP2* protein levels were significantly decreased in R87C/R87C NPCs despite unchanged mRNA levels (Fig. 2B, D; Figure S4A-B). Furthermore, *WAVE1* mRNA levels, which normally increased during neural induction, were reduced in the presence of the variant (Fig. 2B).

Next, we investigated if cell morphology was affected by the variant, as CYFIP2 is involved in WRC-mediated actin polymerization. Electron microscopy revealed cell morphology alteration and lamellipodia impairment in R87C/R87C NPCs (Fig. 2E). Since lamellipodia are critical for neural stem cell migration during development, this result raised the question of whether cell motility was impacted. A high-content imaging cell motility assay confirmed that NPC motility, including speed and displacement, was decreased in the R87C variant cells (Fig. 2F; Supplementary Data 1–4; Figure S5). As a control, treatment with the ROCK inhibitor Y-27632, a negative regulator of NPC motility^22^, similarly reduced NPC speed and displacement. These results indicate that the *CYFIP2* R87C variant impacts gene expression, cytoskeletal organization, and motility in NPCs, processes essential for human cortical development.

### WT and R87C hESC-derived cortical neurons exhibit comparable differentiation efficiency and electrophysiological properties

Following the observations of the impact of the R87C variant in NPCs, we investigated its effect on cortical neurons. Immunostaining confirmed that both R87C/R87C and WT lines were able to generate TUJ1-positive neurons (Fig. 2G), indicating successful neuronal induction. Given this, we proceeded to evaluate neuronal function. Using Multi-Electrode Array (MEA) technology, electrophysiological parameters were similar between WT and R87C/R87C cultures, even after 90 days of differentiation (Fig. 2H; Figure S6). These findings indicate that, under the conditions analyzed, the R87C variant does not overtly impair the ability of cortical neurons to acquire neuronal identity or electrophysiological function.

### Cortical organoids carrying the R87C variant lack NPC marker expression

**Fig. 3:**
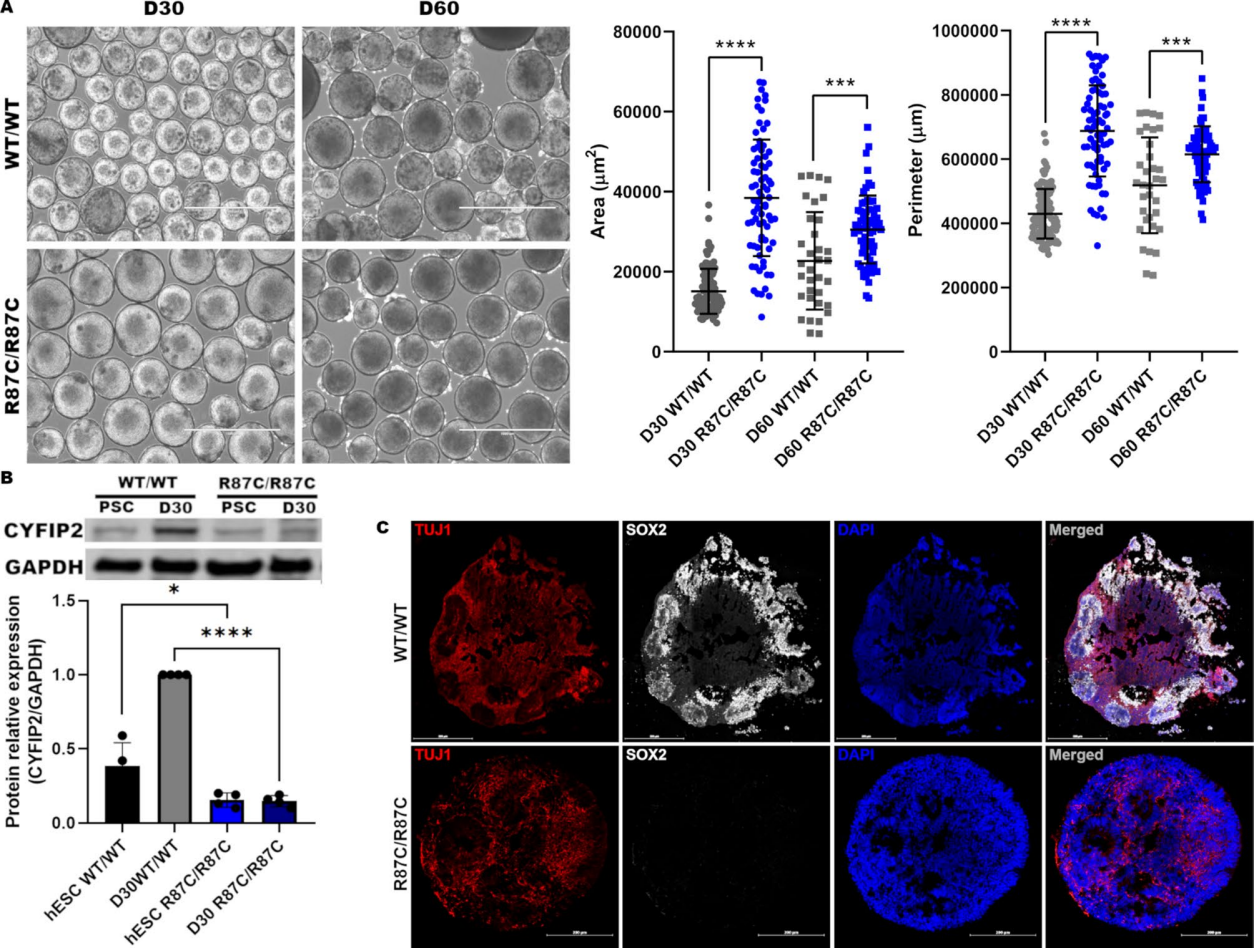
Analysis of R87C/R87C cell line during cortical organoid differentiation. (**A**) Brightfield images of cortical organoids at day 30 and day 60 of differentiation. Scatter plots showing organoid area and perimeter. Each point represents an individual organoid; bars indicate mean ± SD. Scale bar: 1000 μm. ***p ≤ 0.001, ****p ≤ 0.0001; *n* = 3 independent batches. (**B**) Western blot of CYFIP2 in hESC and Day 30 cortical organoids. GAPDH served as housekeeping protein. *n* = 3 independent batches. Statistical analysis: Mean ± SD (*p ≤ 0.05, ****p ≤ 0.0001). Full, uncropped images of this blot are provided in Supplementary Fig. 4. (**C**) Representative immunofluorescence image of Day 30 cortical organoids stained with TUJ1 (red, neuron) and SOX2 (white, NPC). DAPI: blue. Scale bar: 500 (WT/WT) and 200 (R87C/R87C) µm.

Given the monolayer differentiation results, we next employed a three-dimensional model to investigate additional developmental parameters. hESC-derived cortical organoids were analyzed after 30 and 60 days of differentiation. R87C/R87C cortical organoids showed increased size by day 30, and this increase remained significant at day 60 compared to WT organoids (Fig. 3A). On day 30, R87C/R87C organoids exhibited decreased CYFIP2 protein levels compared to WT (Fig. 3B; Figure S4C-D), consistent with the monolayer NPC findings. Immunostaining revealed SOX2 + neural progenitors within rosettes in WT organoids, whereas R87C/R87C organoids lacked SOX2 + cells (Fig. 3C, Figure S7), suggesting NPC loss in these rosette-like structures. Differentiation into cortical neurons appeared unaffected, as their presence remained similar between WT and R87C/R87C organoids (Fig. 3C). Together, these results suggest that the R87C variant disrupts cerebral development by impairing NPC maintenance, potentially leading to downstream effects in later stages.

### hiPSC-derived R87C/WT NPC exhibit reduced CYFIP2 levels and impaired cell motility

**Fig. 4:**
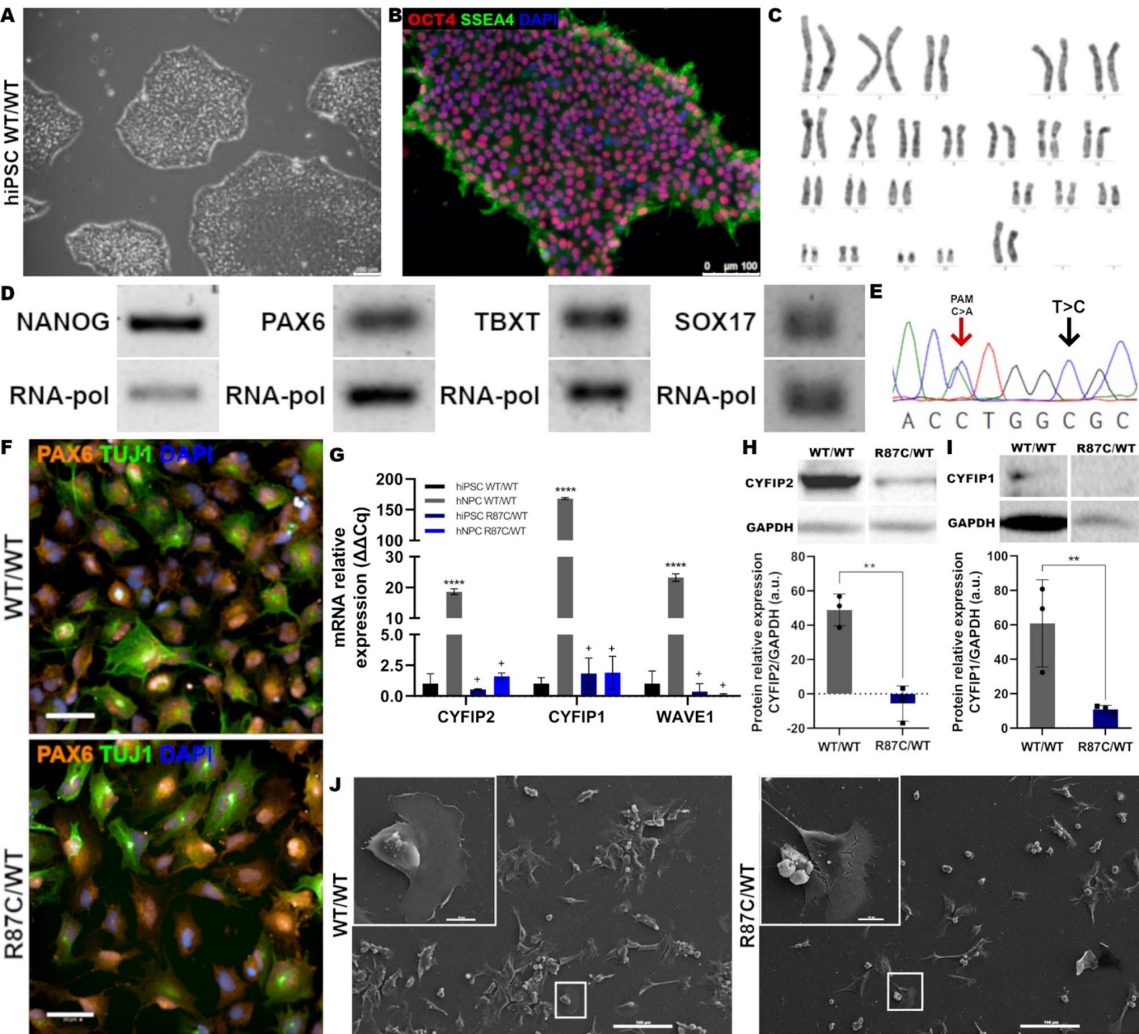
Analysis of hiPSC derived from patient after gene editing and neurogenesis. (A) Representative (**A**) Representative image of a hiPSC colony after gene editing to correct the R87C variant. Scale bar: 100 μm. (**B**) Representative immunofluorescence image identifying pluripotency markers in edited hiPSC. OCT4: red. SSEA4: green. DAPI: blue. Scale bar: 100 μm. (**C**) Representative G-band karyotype showing no chromosomal abnormalities post-gene editing. (**D**) Gel showing pluripotency marker NANOG and germ layer markers PAX6 (ectoderm), TBXT (mesoderm), and SOX17 (endoderm) after induced differentiation. RNApol served as housekeeping gene. (**E**) Electropherogram showing the absence of the C > T mutation on exon 4 of the *CYFIP2* gene post-CRISPR/Cas9 editing, and the presence of the heterozygous silent C > A mutation in the PAM sequence, indicating correction of the affected allele. (**F**) Representative images of hiPSC-derived neural progenitor cells (NPCs) expressing NPC markers TUJ1 (green) and PAX6 (orange). DAPI: blue. Scale bar: 50 μm. (**G**) Expression of *CYFIP2*, *CYFIP1*, and *WAVE1* after NPC differentiation induction. *n* = 3. Statistical analysis: Mean ± SD (hESC WT/WT vs. NPC WT/WT: ****p ≤ 0.0001; other conditions vs. NPC WT/WT: +*p* ≤ 0.0001). (**H**) Western blot analysis of CYFIP2 and (I) CYFIP1 expression in NPCs. GAPDH served as housekeeping protein. Statistical analysis: Mean ± SD (**p ≤ 0.01). (**J**) Representative SEM image showing NPC morphology of WT and R87C cells. Scale bars: 10 (top) and 100 (bottom) µm. Full, uncropped gels and blots corresponding to the panels D, H, and I in this figure are shown in Supplementary Figs. 4 and 7.

An iPSC line carrying the R87C/WT genotype, previously generated^21^, was gene-edited using CRISPR to correct the mutation in the *CYFIP2* gene. These cells maintained pluripotent stem cell morphology in culture and expressed pluripotency markers (Fig. 4A-B and D; Figure S8). Furthermore, the WT cell line exhibited normal karyotypes (Fig. 4C) and differentiated into ectodermal, mesodermal, and endodermal cells upon direct induction of germ layer differentiation (Figs. 4D and 87). Sanger sequencing verified correction of the mutation in exon 4 of the *CYFIP2* gene along with the introduction of a silent mutation in the PAM sequence to prevent re-cutting (Fig. 4E), indicating successful donor DNA-mediated repair. Collectively, these results established an isogenic pair of isogenic pair of pluripotent cell lines to study the effects of the R87C variant during neurogenesis.

To evaluate the impact of the R87C variant in a patient-derived cell line, NPC differentiation was induced in the isogenic hiPSC pair. Simiilarly to the hESC differentiation, NPC differentiation was unaffected (Fig. 4F). Analysis of *CYFIP2* and *CYFIP1* expression revealed reduced mRNA and protein levels in R87C/WT NPCs (Fig. 4G-I; Figure S4E-F), accompanied by reduced *WAVE1* mRNA levels (Fig. 4G). hiPSC-derived R87C/WT NPCs also exhibited altered cell morphology and lamellipodia formation as analyzed by electron microscopy (Fig. 4J). These data corroborate findings from the hESC model, suggesting a pronounced impact of the CYFIP2 R87C variant on neurodevelopment at early stages.

## Discussion

Leveraging hPSC-derived human neuronal models, our investigation partially corroborated key findings from the mouse model^17^, specifically that the R87C variant leads to a decrease in CYFIP2 protein levels and impacts cortical growth. A notable distinction, however, was the impact of the variant on mRNA levels. While the R87C mutation in mice does not appear to alter *Cyfip2* mRNA stability in the cortex or hypothalamus^17^, our human patient-derived cells exhibited a significant failure in transcript accumulation. This finding is of interest given that CYFIP2 is a neuron-dominant gene^23^, suggesting that an increase in *CYFIP2* transcripts is a typical physiological requirement during the transition to a neural identity. Our data indicates that the presence of the R87C variant may hinder this expected surge in human cells.

These observations raise the possibility that the R87C variant could be impacting broader post-transcriptional regulation. In support of this hypothesis, we observed decreased mRNA and protein levels of *CYFIP1* and *WAVE1*, whereas the mouse model reportedly showed only a decrease in Wave1 protein without impacting Cyfip1 levels. This divergence is potentially significant because, while *CYFIP2* mRNA is known to be FMRP-regulated, *CYFIP1* and *WAVE1* transcripts are reportedly not^24^. Furthermore, the CYFIP1 gene remained unaffected by our CRISPR/Cas9 editing (Figure S3). This could suggest a human-specific involvement of CYFIP2 in wider post-transcriptional regulatory networks. As previously noted by Lee et al. (2020), R87 variants may impair stress granule formation and interact with other RNA-binding proteins (RBPs)^25^. To our knowledge, this study provides a novel description of this possible transcriptomic modulation in a human model for this variant. Future studies with RNA-seq and polysome profiling could clarify the variant’s post-transcriptional disruptions and their relevance to the severe clinical phenotypes observed in patients.

Our 2D monolayer cultures provided valuable initial insights, offering a baseline for cellular function. Consistent with the mouse model^17^, where R87C animals did not show spontaneous seizures, we detected no electrophysiological differences in monolayer-cultured cortical neurons. This suggests the R87C variant may not induce acute functional deficits in a simplified 2D environment. However, since patients with this variant do experience seizures, this highlights a critical limitation of simplified models and underscores the necessity of 3D models to unravel complex phenotypes that manifest in more physiologically relevant contexts. Cortical organoids proved invaluable for exploring these complex phenotypes. R87C variant-derived organoids were significantly larger on both day 30 and day 60 compared to WT. Although reduced neural progenitor cell (NPC) pools are often associated with smaller organoids and microcephaly^26^, our observations align with findings reported in another neurodevelopmental condition modeled using a similar organoid approach^27^. For example, Urresti et al. (2021) showed that 16p11.2 deletion organoids were significantly larger at 1- and 3-month stages despite a marked depletion of SOX2 + and PAX6 + progenitors^28^. This demonstrates that early increases in organoid size can coexist with disruptions in progenitor maintenance and may reflect altered neurogenic trajectories rather than preserved proliferative capacity. Although we did not perform additional analyses to evaluate growth rates across developmental stages, the similarities between our results and these previously described neurogenic alterations suggest that the R87C variant may likewise influence early developmental pathways captured by cortical organoids. This observation in the organoid model reinforces its advantage in mimicking in vivo development and complex disease manifestations^18,29^. Further studies are needed to investigate how the absence of these crucial cells impacts the overall growth and development of the organoids.

Regarding cell morphology and motility, the R87C variant led to a less-spread NPC morphology and altered lamellipodia formation, suggesting downstream effects on the cytoskeleton and cell-matrix interactions. This is supported by the decreased cell speed and displacement of R87C/R87C cells, implying an impact on cytoskeleton organization given lamellipodia’s role in motility. This contrasts with Begeman and collaborators (2021), who showed that while the formation of actin-rich structures (dorsal ruffles) was impacted by the R87C variant in patient fibroblasts, broader actin disorganization was not observed^15^. This discrepancy may be due to lower CYFIP2 expression in fibroblasts versus neurons. Our study addresses this by showing that patient-derived iPSCs corroborate the hESC model findings, including decreased CYFIP2 levels and impacts on morphology and lamellipodia. Choi et al. further showed that, in triple-negative breast cancer cell lines, a decrease in CYFIP2 levels could increase filopodia formation and actin accumulation, and impact cell migration and adherence^30^. Thus, the decreased CYFIP2 levels with the R87C variant may cause an imbalance in actin polymerization between lamellipodia and filopodia.

In conclusion, this study provides the first description of the R87C variant’s impact on human neuronal models in vitro. Further research is needed to delve deeper into its neurodevelopmental impacts and the translation to patient symptoms. Crucially, understanding the molecular and cellular effects of the R87C variant can guide the search for root-cause treatments, potentially improving patients’ quality of life.

### Methods

#### Ethical committee and patient recruitment

The study was approved by the Research Ethics Committee (CEP) of Fiocruz and the Brazilian National Research Ethics Committee (CONEP), under opinion number 3.856.868, CAAE: 16918819.7.0000.5248. All research was performed in accordance with the relevant guidelines, regulations, and the Declaration of Helsinki. The hiPSC line SP1C8 was generated in a previous study by our group^21^ and is stored in our institutional repository. The line is available upon reasonable request from the corresponding author. The collection and processing of the sample were authorized by the parents in an Informed Consent Form (ICF), and it was used in full compliance with all ethical and legal regulations.

#### Cell culture

In this study, the hESC H9 (WiCell, WA09)^31^ and the hiPSC SP1C8^21^ cell lines were used. Cells were cultivated on Vitronectin (Invitrogen) or Geltrex (Gibco)-coated plates in StemFlex media (Gibco). Cells were passaged with ReLeSR (Stem Cell Technologies) or Accutase (Gibco) and re-plated in StemFlex. When cells were passaged with Accutase, the media was supplemented with 10 µM Y-27632 (Sigma) or 2 µM thiazovivin (Sigma) as ROCK inhibitors, which was removed the following day. For freezing, cells were dissociated with ReLeSR (Stem Cell Technologies) or Accutase (Gibco) and resuspended in a 90% StemFlex 10% DMSO solution or CryoStor10 (Stem Cell Technologies). When dissociated with Accutase, cells were frozen at a concentration of 1 × 10^6^ cells/vial. Cells were kept under antibiotic-free conditions. For hESC line, passages above #80 were used during experimentation. For hiPSC line, passages above #12 were used.

### CRISPR/Cas9 gene editing and off-target selection and sequencing

Isogenic hESC lines (R87C mutation introduction) and hiPSC lines (R87C/WT mutation correction) were generated using CRISPR/Cas9. A gRNA targeting the mutation site was designed (Figure S1); for hiPSC correction, the gRNA included the C > T mutation to specifically target the mutated allele. The gRNA was cloned into pX458 (Addgene #48138). Cells were by transfection with 3 µg plasmid pX458 (Addgene#48138) and 5 µg donor single-strand DNA using the Neon Transfection system (Invitrogen), per manufacturer’s instructions. After 24 h, GFP-positive single cells were sorted using a WOLF G1 Cell sorter (NanoCellect). Clones were screened after 10 days by HinP1I restriction enzyme (NEB). Positive clones were confirmed by sequencing. For off-target selection, genes were selected from a list provided by the Benchling program. Genes were selected based on an off-target score above 1.0 and with gRNA alignment in exonic regions of coding genes. The *CYFIP1* gene was also sequenced, despite not being identified as an off-target. Samples were prepared and sent to sequencing by GoGenetic (Curitiba, Brazil). Primers used for sequencing are described in Supplementary information. No Next Generation Sequencing (NGS) data was generated for this study. Sequencing efforts were restricted to targeted Sanger sequencing of PCR products from pre-selected genomic regions for confirmation of CRISPR/Cas9 edits and off-target analysis.

### Characterization of hESC clones after CRISPR/Cas9 editing

To assess pluripotency characteristics after CRISPR/Cas9 editing, hESCs were plated for immunostaining and RNA extraction for pluripotency marker analysis. To evaluate differentiation capacity, cells were induced to spontaneous differentiation into the three germ layers, as previously described^32^. First, cells under 80% confluency were incubated with EB media (DMEM/F12 supplemented with 20% knockout serum, 0.2% β-mercaptoethanol, and 1X non-essential amino acids). On the following day, cells were washed with 1X DPBS (Gibco), detached from the plate using a cell scraper, transferred to a new plate containing EB media, and kept under rotation (90 rpm) at 37 °C and 5% CO₂ for 7 days, with media changes every other day. After this period, EBs were collected for RNA extraction.

### Characterization of hiPSC clones after CRISPR/Cas9 editing

To characterize hiPSCs after CRISPR/Cas9 editing, cells were plated for immunostaining and RNA extraction for pluripotency marker analysis. To assess differentiation capacity, cells were induced toward each germ layer, as previously described^21^. For ectodermal differentiation, the PSC Neural Induction Kit (Gibco) was used according to the manufacturer’s instructions. Briefly, 2.5 × 10⁶ cells were plated into a Geltrex^®^-coated (Gibco) 6-well plate in StemFlex™ media (Gibco) supplemented with 2 µM thiazovivin (Sigma) (ROCK inhibitor). On the next day (D1), the media was replaced with Neural Induction Media (NIM) (Neurobasal medium supplemented with 1X Neural Induction Supplement (NIS)). Media was replaced on days 3, 5, and 7 (with double volume on D5 and D7). On day 8, cells were collected for RNA extraction.

For mesodermal differentiation, 2.5 × 10⁶ cells were plated into a Geltrex^®^-coated (Gibco) 24-well plate in StemFlex™ media (Gibco) supplemented with 2 µM thiazovivin (Sigma) (ROCK inhibitor). The media was changed over the next two days to reach maximum confluency. On day 0, cells were induced to mesoderm using basal media (RPMI supplemented with 1X B27 minus insulin) supplemented with 12 µM CHIR99021. On the following day (D1), cells were collected for RNA extraction,

For endodermal differentiation, 1 × 10⁶ cells were plated into a Geltrex^®^-coated (Gibco) 24-well plate in StemFlex™ media (Gibco) supplemented with 2 µM thiazovivin (Sigma) (ROCK inhibitor). On the next day, the media was changed to remove the ROCK inhibitor. On day 0, cells were induced using basal media (RPMI supplemented with 1X B27 minus insulin, 1% L-glutamine, and 1% non-essential amino acids) supplemented with 3 µM CHIR99021 and 50 ng/mL Activin A. On day 2, the media was replaced with basal media supplemented with 50 ng/mL Activin A. On day 4, cells were collected for RNA extraction.

### Karyotype of hPSC clones

For chromosome analysis, samples were prepared as previously described^21^. Briefly, cells were arrested in metaphase by treatment with colchicine (0.32 µg/ml), then subjected to hypotonic treatment with 57 mM potassium chloride for 10 min at 37 °C, followed by fixation. Fixation was performed sequentially using Carnoy’s solution (methanol: acetic acid): first with a 3:1 ratio for 10 min at − 20 °C, and then washed twice with a 2:1 ratio. Finally, G-banding was performed to visualize and analyze the chromosomes.

### hESC neurogenic differentiation in monolayer

For hESC-derived cortical neuron differentiation, an adapted protocol from Ahn et al. (2021)^32^ was used. To generate neural progenitor cells (NPCs), 3 × 10^6^ cells were resuspended on Day 1 in N2B27 media (DMEM/F12, 1X N2, 1X B27, 150 µM ascorbic acid, 0.5% Glutamax, 1% penicillin/streptomycin; all Gibco) supplemented with 1 µM dorsomorphin, 10 µM SB431542, 3 µM CHIR99021, 0.5 µM purmorphamine, and 10 µM Y-27632. On Day 2, media was partially replaced with N2B27 with 1 µM dorsomorphin, 10 µM SB431542, 3 µM CHIR99021 and 0.5 µM purmorphamine, without Y-27632. On Day 6, media was changed to NPC maintenance medium (N2B27 with 3 µM CHIR99021 and 0.5 µM Purmorphamine). EBs were plated on Matrigel (Corning) on Day 8, and dissociated with Accutase and expanded 1:5 on Day 10 to obtain NPCs. NPCs were kept under culture in NPC maintenance media and Matrigel-coated plates. All experiments were conducted with NPCs over passage 4. For cortical neuron differentiation, NPCs were plated in poly-ornithine/laminin-coated wells in NPC medium. On Day 1, medium was changed to N2B27 supplemented with 10 µg/ml BDNF and 10 µg/ml GDNF. On Day 14, neurons were passed onto new coated plates using 1:1 Accutase: Accumax for 20 min at 37 °C, then strained, counted, and plated for assays. Cortical neurons were maintained for a minimum of 30 days, up to 60 days for maturation.

### hiPSC NPC differentiation and culture

To generate hiPSC-derived NPCs, the PSC Neural Induction Kit (Gibco) was used according to the manufacturer’s instructions, as described above (“*Characterization of hiPSC clones after CRISPR/Cas9 editing*” sub-section). On day 8, cells were re-plated using Accutase (Gibco) onto Geltrex-coated plates and cultivated in Neural Expansion media (NEM) (1:1 DMEM/F12:Neurobasal media, supplemented with 1X NIS). All experiments were conducted with NPCs over passage 2.

### Generation of the cortical organoids

Cortical organoids were generated following Trujillo et al. (2019)^27^. Briefly, on Day 1, 2 × 10^6^ cells were resuspended in StemFlex (Gibco) supplemented with 1 µM dorsomorphin, 10 µM SB43154, and 10 µM Y-27632, then rotated (90 rpm) to form EBs. On Day 4, media was changed to basal media (DMEM/F12, 1X N2, 1X B27, 1X non-essential amino acids, 100 U/ml penicillin/streptomycin) supplemented with 1 µM dorsomorphin and 10 µM SB43154. Subsequent media changes included: Day 11 – basal media with 20 ng/ml bFGF; Day 18 – basal media with 20 ng/ml bFGF and 20 ng/ml EGF; Day 25 – basal media with 10 ng/ml BDNF, 10 ng/ml GDNF, 10 ng/ml NT-3, 200 µM ascorbic acid, and 1µM dibutyl cAMP. From Day 32, organoids were maintained in basal media only.

### RNA extraction, cDNA reaction, and PCR

Total RNA was extracted from cells or organoids using the Trizol method. Briefly, samples were lysed in 500 µl Trizol, followed by chloroform extraction and phase separation. cDNA synthesis was performed using SuperScript IV VILO (Invitrogen) or RT Improm-II (Promega) kits according to manufacturers’ instructions. PCR for pluripotency and germ layer markers was performed using the NAT kit (IBMP), with 10 pmol of each primer and 1 µl of sample. PCR products were separated on a 2% agarose gel stained with SyBR Safe DNA Gel stain (Invitrogen) and visualized. For qPCR, GoTaq qPCR master mix kit (Promega) was used, and reactions were performed in QuantStudio™ 5 (Thermo Fisher Scientific). Primers used in all PCR reactions are described in Supplementary information.

### Protein extraction and western blot

For protein extraction, cells were lysed in lysis buffer (25 mM Tris-HCl pH 8.0, 1 mM EDTA, 1% NP-40, 150 mM NaCl, protease inhibitors) for 10 min at 4 °C, followed by sonication (15 cycles of 30 s on/off) and centrifugation (20,000 x *g* for 20 min at 4 °C). Supernatants were collected, and protein concentration was determined using the BCA Protein Assay (Thermo Fisher Scientific). For Western blot, proteins were separated by 12% SDS-PAGE and transferred to PVDF membranes using a Trans-Blot Turbo Semi-dry transfer system (Bio-Rad, high molecular weight program). Membranes were blocked with buffer (PBS 0.1% Tween 20, 5% milk, 3% BSA) for 1 h, then incubated with primary antibodies (supplementary material) overnight at 4 °C with rotation. After incubation with infrared-conjugated secondary antibodies, membranes were visualized on an Odyssey^®^ XF (Li-Cor Biosciences) and band intensities quantified using Empiria Studio^®^ (Li-Cor Biosciences) software.

### Cell tracking assay

NPC motility was assessed via cell tracking assay using the Operetta CLS high-content imaging system (PerkinElmer). Neural progenitor cells were plated into glass-bottom 96-well plates and imaged at 50% confluency. Plates were maintained at 37 °C and 5% CO2 in the Operetta hardware, capturing digital phase contrast images every 2.5 min for 1 h with a 20X objective. Analysis was performed with Harmony software (v. 4.9), where cells were identified by their cytoplasm to calculate kinetic and tracking properties (Supplementary information). Y-27632 ROCK inhibitor, a known negative regulator of NPC motility^22^, served as a negative control.

### Microscopy assays

For immunofluorescence, cells were fixed with 4% PFA for 15 min at RT, permeabilized with 0.5% Triton X-100 in PBS for 15 min, and blocked with 5% BSA in PBS for 1 h. Primary antibodies (supplementary material) were incubated overnight at 4 °C, followed by secondary antibodies for 1 h at RT. For organoid immunostaining, organoids were fixed with 4% PFA for 15 min at RT under rotation (90 rpm), then dehydrated in 30% sucrose solution overnight at 4 °C with rotation. Organoids were then embedded in OCT resin (Sakura Finetek), frozen at −80 °C, and sectioned into 10 µM slices for subsequent immunostaining as described for cells. For scanning electron microscopy (SEM), cells were plated onto glass coverslips coated with Geltrex (Gibco), then fixed with a 25% glutaraldehyde/0.2 M sodium cacodylate solution (aqueous) for 1 h in room temperature. Samples were dehydrated through a series of successive methanol steps, then coated with gold particles, and images were acquired using a JSM-6010 Plus/LA scanning electron microscope.

### MEA analysis

Cortical neuron electrophysiology was analyzed using a Maestro MEA system (Axion Biosystems), where Day 14 neurons were plated on Lumos MEA 48-well plates (Axion Biosystems). Recordings were collected from Day 15 to Day 94 of differentiation (10-minute recordings, 37 °C, 5% CO2) using AxIS Software Spontaneous Neural Configuration (Axion Biosystems) after a 2-minute plate equilibration. Electrodes were deemed active if they recorded ≥ 5 spikes/min. Neuronal activity was assessed by calculating the firing rate, defined as the number of spikes (action potentials) emitted over time for each electrode individually. In this study, it was considered the weighted mean firing rate, which accounts exclusively for readings from active electrodes, providing a more accurate estimation of overall network activity. Weighted firing rate analysis included only recordings above 2 Hz. Oscillatory activity was assessed through burst firing, defined as sequences of spikes emitted in rapid succession, separated by quiescent intervals, occurring synchronously across all active electrodes. Bursts were identified by an interspike interval (ISI) threshold of ≥ 5 spikes and a ≤ 100 ms maximum ISI.

### Statistical analysis

Statistical analysis was performed using GraphPad Prism software (v.8.0.1). Data were analyzed by one-way or two-way ANOVA, as appropriate for the dataset, followed by Tukey’s multiple comparisons test, with a 95% confidence interval. Statistical significance was set at *p* ≤ 0.05.

## Supplementary Information

Below is the link to the electronic supplementary material.

Supplementary Material 1

Supplementary Material 2

Supplementary Material 3

Supplementary Material 4

Supplementary Material 5

Supplementary Material 6

## Data Availability

All data reported in this paper will be shared by the lead contact upon request. This paper does not report original code. Any additional information required to reanalyze the data reported in this paper is available from the lead contact upon request.

## Declarations

### AI use statement

During the preparation of this work the authors used Google Gemini (v. 2.5) in order to improve readability and language. After using this tool/service, the authors reviewed and edited the content as needed and take full responsibility for the content of the publication.
