# Supplementary material for: Impact of the CYFIP2 R87C variant in a human neuronal model in vitro
Source: Sci Rep. 2026 Mar 18;16:13967. doi: 10.1038/s41598-026-44176-2 (PMC13133249; doi:10.1038/s41598-026-44176-2)
Supplement: Supplementary file 5 — Supplementary Material 5 [file 41598_2026_44176_MOESM5_ESM.pdf]

# Impact of the CYFIP2 R87C variant in a human neuronal model in vitro

Zaboroski-Silva et al – Supplementary information

**Supplementary table 1: List of antibodies used for immunofluorescence (IF) and Western blot (WB) assays**

| Marker | Assay | Protein | Brand | Catalog | Host | Dilution |
|---|---|---|---|---|---|---|
| Pluripotency | IF | NANOG | Santa Cruz | sc-293121 | Mouse | 1:200 |
| Pluripotency/ NPC | IF | SOX2 | eBioscience | 14-9811-82 | Rat | 1:1000 |
| NPC | IF | PAX6 | Invitrogen | 901301 | Rabbit | 1:1000 |
| Immature neuron | IF | TUJ1 | Invitrogen | 801201 | Mouse | 1:1000 |
| Matture neuron | IF | MAP2 | Abcam | ab5392 | Chicken | 1:1000 |
| Glia | IF | GFAP | Abcam | ab4674 | Chicken | 1:1000 |
| - | WB | CYFIP1 | Gentex | GTX122467 | Rabbit | 1:500 |
| - | WB | CYFIP2 | Abcam | ab95969 | Rabbit | 1:1000 |
| - | WB | GAPDH | Santa Cruz | sc-47724 | Mouse | 1:10000 |

**Supplementary table 2: List of primers used for sequencing, PCR and qPCR**

| Gene | *Foward* 5'-3' | *Reverse* 5'-3' | Amplicon |
|---|---|---|---|
| *POU5F1* (OCT4) | ATGCATTCAAACTGAGGTGCCTGC | AACTTCACCTTCCCTCCAACCAGT | 192 bp |
| *NANOG* | ACCAGAACTGTGTTCTCTTCCACC | CCATTGCTATTCTTCGGCCAGTTG | 200 bp |
| *NESTIN* | GGGAAGAGGTGATGGAACCA | AAGCCCTGAACCCTCTTTGC | 64 bp |
| *PAX6* | CAGCACCAGTGTCTACCAACCA | CAGATGTGAAGGAGGAAACCG | 62 pb |
| *MSX1* | AGGACCCCGTGGATGCAGAG | GGCCATCTTCAGCTTCTCCAG | 300 bp |
| *TBXT* (BRACH) | AAAGAGATGATGGAGGAACCCGGA | AGGATGAGGATTTGCAGGTGGACA | 108 bp |
| *SOX17* | AGTGACGACCAGAGCCAGAC | CCTTAGCCCACACCATGAAA | 214 bp |
| *CYFIP1* | AGTTTGTGGCGCTTTGTCTG | CCTGCTGTGGGGTGAGAAC | 291 bp |
| *CYFIP2* | AGTGAGGATCTGGGGGCTTT | ACCTGCTTTACTCCCTACCAC | 272 bp |
| *WAVE1* | AAGCGCCATCCATCAACC | ACGGCGAGACAGGATGGT | 162 bp |
| *DHODH* | TCTGTAGGCCGAGTTCCCAT | CAAGCCACACATGACCTCAC | 191 bp |
| *PLEKHA4* | CATGGCTTCTGAAGTCTAACCAC | AGTCAAGTGACAGAGGGTGGC | 187 bp |
| *UBE2G2* | GTGGCCCGGAAGCAGTC | ACGTTTGTACTCGGCCATCA | 130 bp |
| *UBXN11* | CAGCCCCCAATAAAGCAC | CCACTTAGCCCTTCTTGGGA | 215 bp |
| *PKMYT1* | CTTTCTGTCCCCATCCCGTC | CCATGGCCAGCCACTATCTC | 200 bp |

| | | | |
|---|---|---|---|
| *GAPDH* | GGCGATGCTGGCGCTGAGTAC | TGGTTCACACCCATGACGA | 149 bp |
| *POLR2A* (RNApol) | TACCACGTCATCTCCTTTGATGGCT | GTGCGGCTGCTTCCATAA | 186 bp |

**Supplementary table 3: Cell tracking assay workflow on Harmony software**

(A) Input image
- Flatfield correction: None
- Brighfield correction: No
- Stack processing: individual planes
- Create global image: Yes
- Min. Global Binning: Dynamic

(B) Find cells
- Channel: Digital Phase contrast
- ROI: None
- Method: P
    - Output population: Cells

(C) Select population
- Population: Cells
- Method: Common Filters
    - Remove Border objects: Yes
- Output population: Cells Selected

(D) Track Objects
- Population: Cells Selected
- Region: Cell
- Method: Standard
    - Output population: Tracked cells selected

(E) Calculate Kinetic Properties
- Population: Tracked cells selected
- Method: Standard
    - Current Speed: Yes (μm/s)

(F) Calculate Track Properties
- Population: Tracked cells selected
- Method: Standard
    - Accumulated distance: Yes (μm)
    - Displacement: Yes (μm)
    - Speed: Yes (μm/s)
    - Displacement X: Yes (μm)
    - Displacement Y: Yes (μm)

(F) Calculate Track Properties (2)
- Population: Tracked cells selected
- Method: Time Aggregation
    - Current Speed: Yes (μm/s)
    - Current Displacement X: Yes (μm)
    - Current Displacement Y: Yes (μm)

Define results
- Method: List of Outputs
    - Population: Tracked cells selected
        - Number of objects: Yes
        - Apply to all: Mean

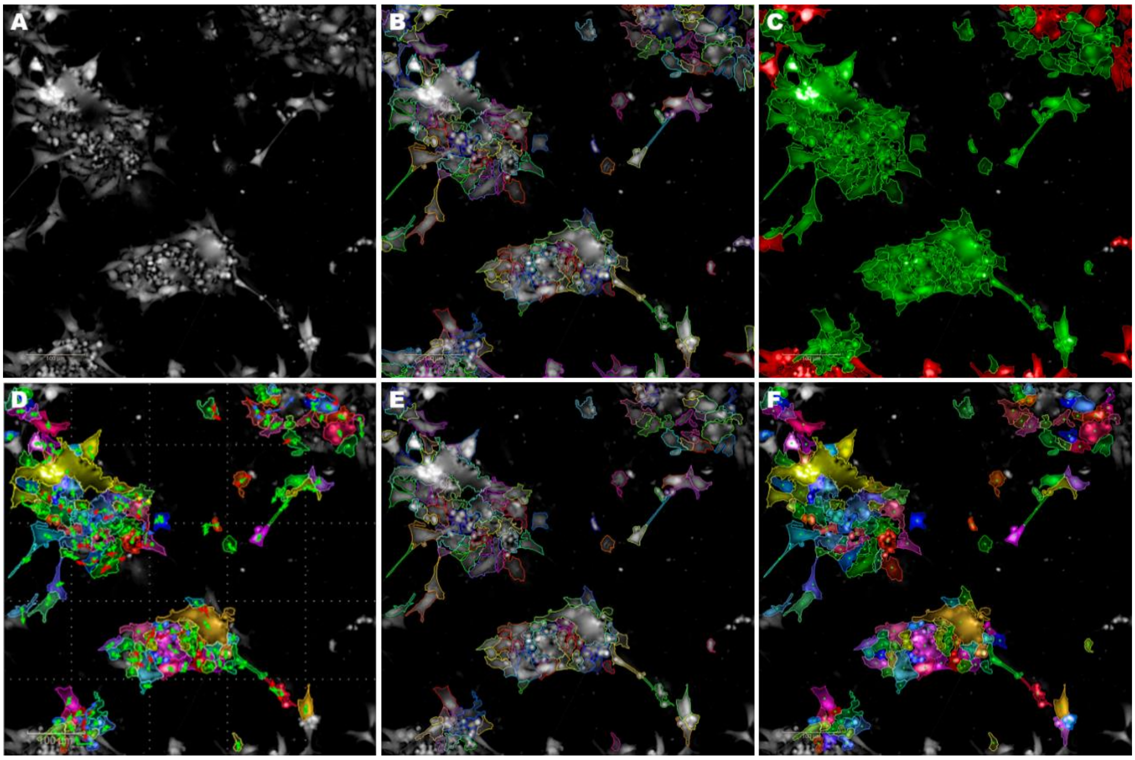


**Operetta HCS Analysis Layout for Cell Tracking Assay.** NPC cell motility was determined using Operetta CLS and Harmony Software 4.9 (PerkinElmer) through a sequential analysis of 4 images per well (20X objective) for 1 hour, with photos taken every 2.5 minutes (25 timepoints). n=3 for the Y-27632 treatment; n=4 for the "No treatment" condition.

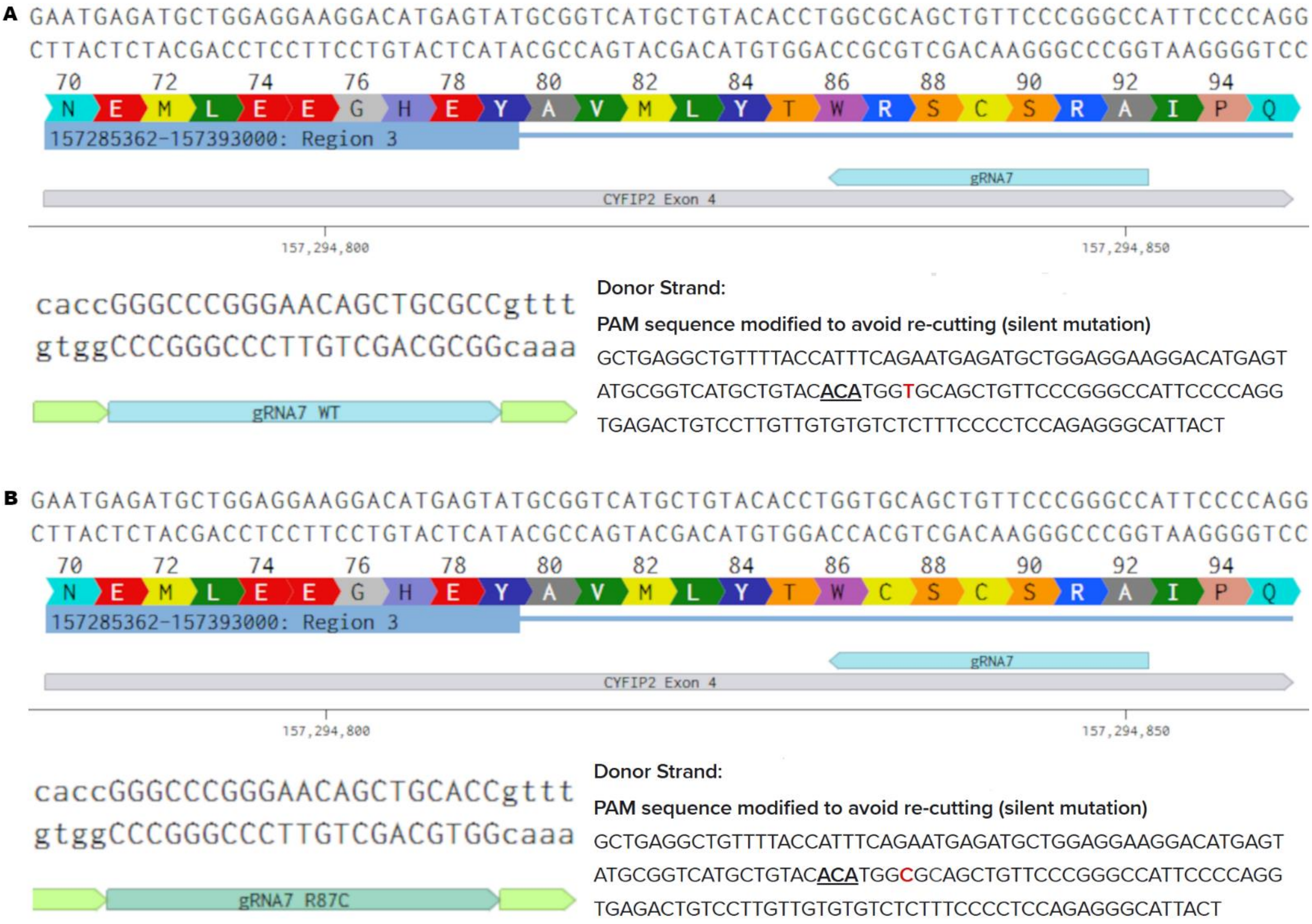


**Supplementary Fig. 1: gRNA and Donor DNA Design.** (A) Exon 4 of the CYFIP2 gene, with the gRNA targeting the R87 region for the introduction of a mutation in the hESC cell line. The gRNA WT had overhang arms introduced into the sequence for cloning into the pX458 plasmid. The Donor DNA contained the C>T mutation (red) and a silent mutation in the PAM sequence (bold and underlined) to prevent re-cutting. (B) Exon 4 of the CYFIP2 gene, with the gRNA targeting the R87C region for the correction of the mutation in the hiPSC cell line. The gRNA R87C had overhang arms introduced into the sequence for cloning into the pX458 plasmid. The Donor DNA contained the T>C correction (red) and a silent mutation in the PAM sequence (bold and underlined) to prevent re-cutting.

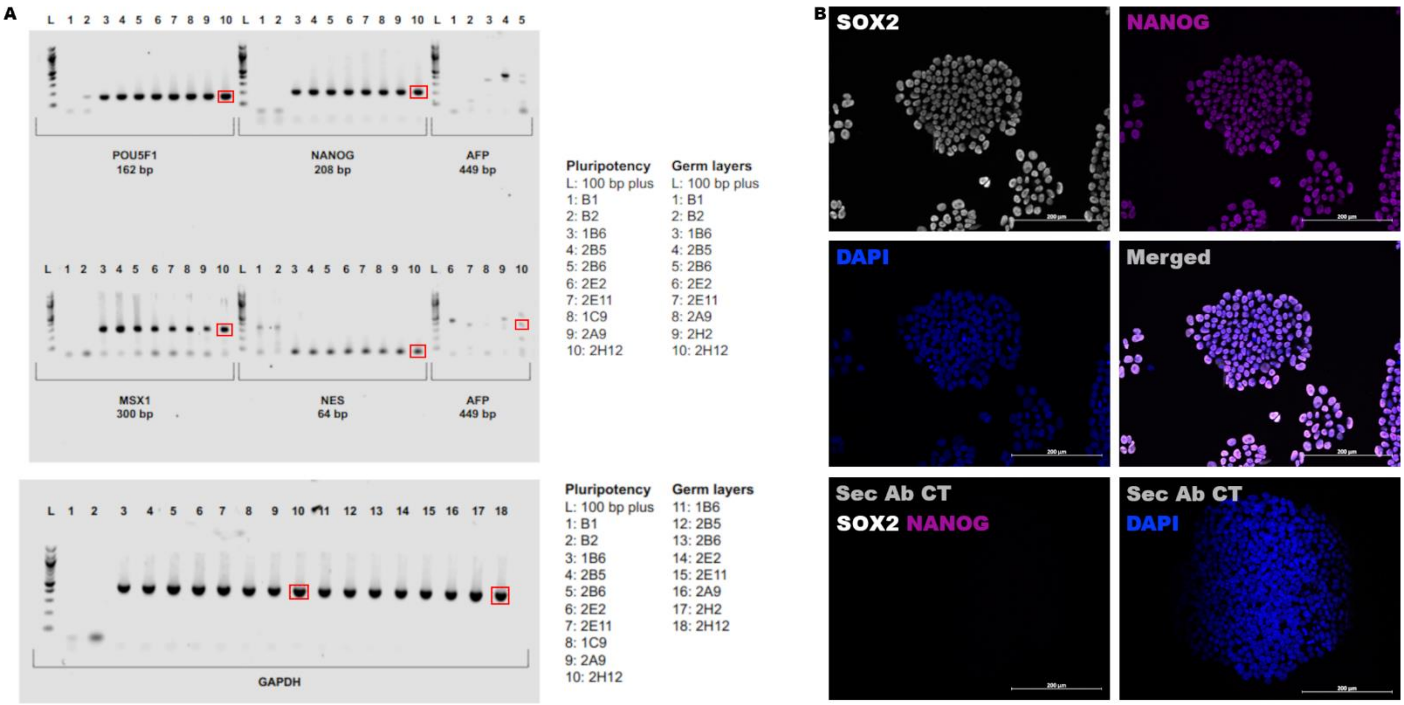


**Supplementary Fig. 2 (Above): Expanded hESC Characterization after CRISPR/Cas9 Gene Editing.** (A) Agarose gel showing the analysis of pluripotency and germ layer marker expression in different clones after gene editing. RNA derived from cells before (Pluripotency) and after undergoing EB formation and spontaneous differentiation (Germ layers) were analyzed using RT-PCR. Each lane represents a different clone generated after CRISPR/Cas9 editing. *POU5F1 and NANOG*: pluripotency markers*. NESTIN: ectoderm marker. MSX1: mesoderm marker. AFP: endoderm marker. GAPDH*: housekeeping gene. Ladder: 100 bp plus. The bands used in Figure 1 are outlined with a red line. (B) Separate channels showing the expression of pluripotency markers in the mutated clone. Sec Ab CT: cells incubated with only the secondary antibody as a control for nonspecific staining.

**Supplementary Fig. 3 (Below): Off-Target Sequencing for hESC and hiPSC Characterization after CRISPR/Cas9 Gene Editing.** Sanger sequencing of the hESC off-target genes: (A) *UBXN11*, (B) *UBE2G2*, (C) *PKMYT1*, and (D) *PLEKHA4*, and the hiPSC off-target gene (E) *DHODH*, showing no alteration after *CYFIP2* gene editing. (F) Sanger sequencing of the *CYFIP1* gene after *CYFIP2* gene editing on hESC cell line, showing no alteration.

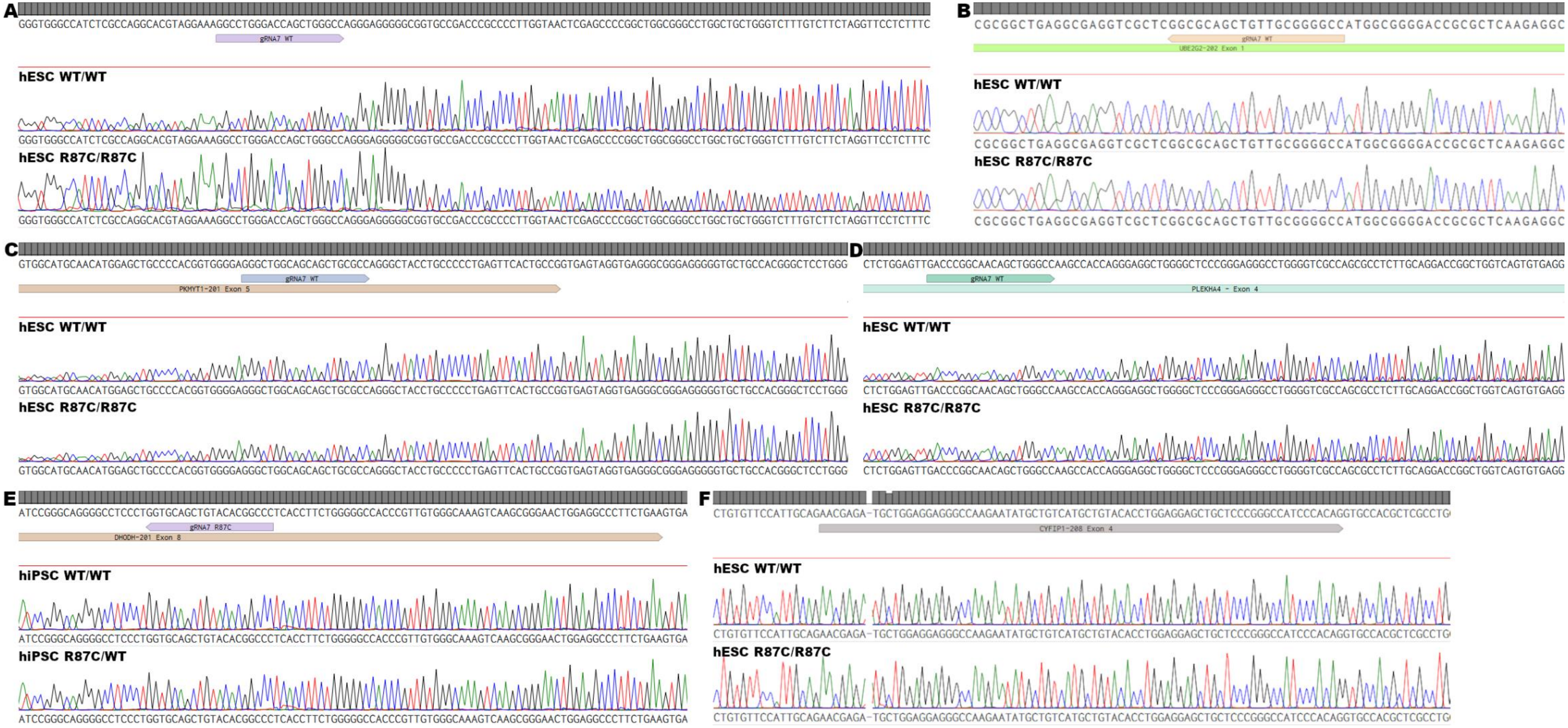
A
gRNA7 WT
hESC WT/WT
hESC R87C/R87C
B
gRNA7 WT
UBE2G2-202 Exon 1
hESC WT/WT
hESC R87C/R87C
C
gRNA7 WT
PKMYT1-201 Exon 5
hESC WT/WT
hESC R87C/R87C
D
gRNA7 WT
PLEKHA4 - Exon 4
hESC WT/WT
hESC R87C/R87C
E
gRNA7 R87C
DHODH-201 Exon 8
hiPSC WT/WT
hiPSC R87C/WT
F
CYFIP1-208 Exon 4
hESC WT/WT
hESC R87C/R87C

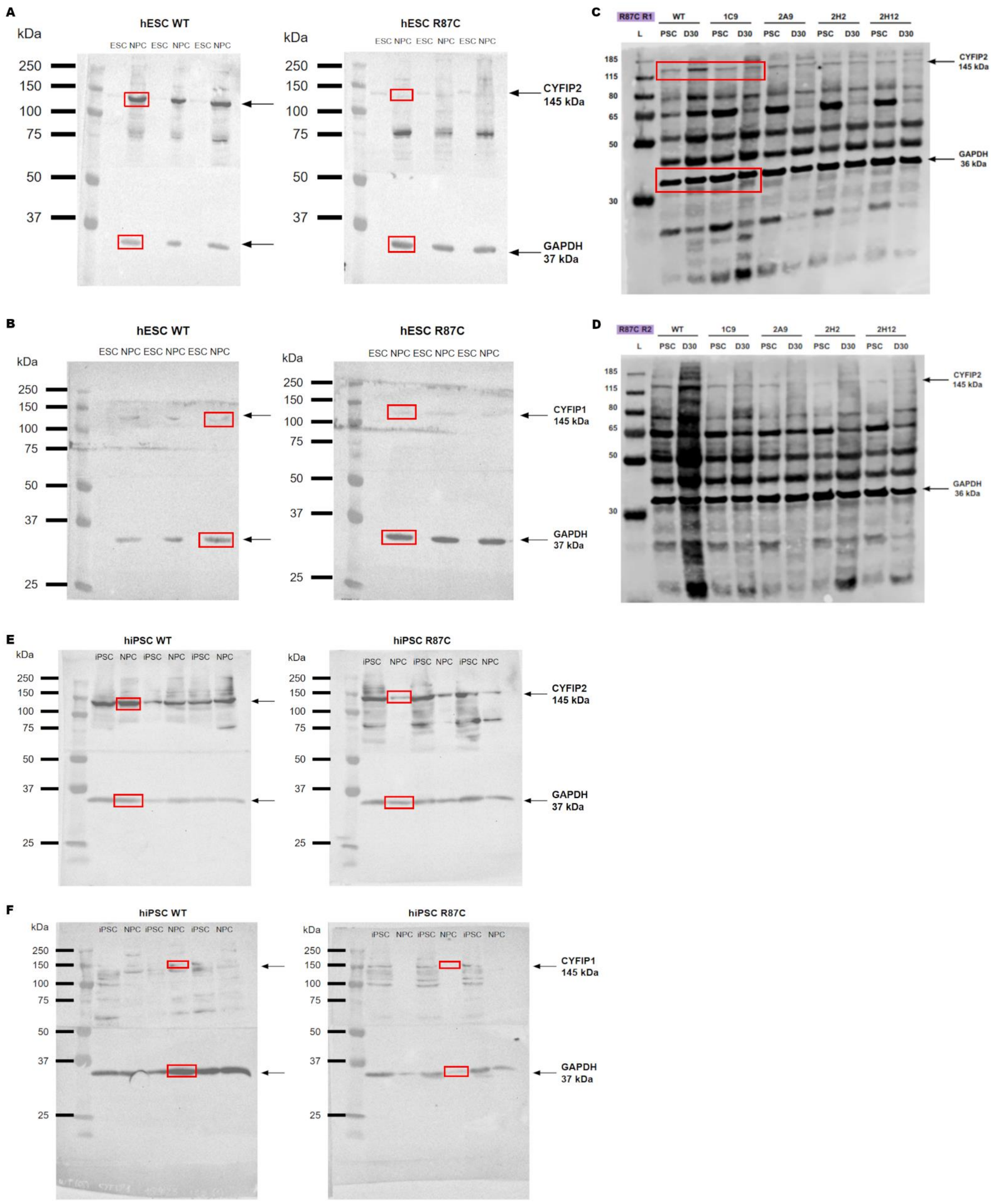


**Supplementary Fig. 4: Expanded Western Blot Membranes of CYFIP2 and CYFIP1.** Western blot membranes of CYFIP2 and CYFIP1 for (A, B) hESC-derived NPCs, (C, D) cortical organoids, and (E, F) hiPSC-derived NPCs. The bands used in Figures 2-4 are outlined with a red line.

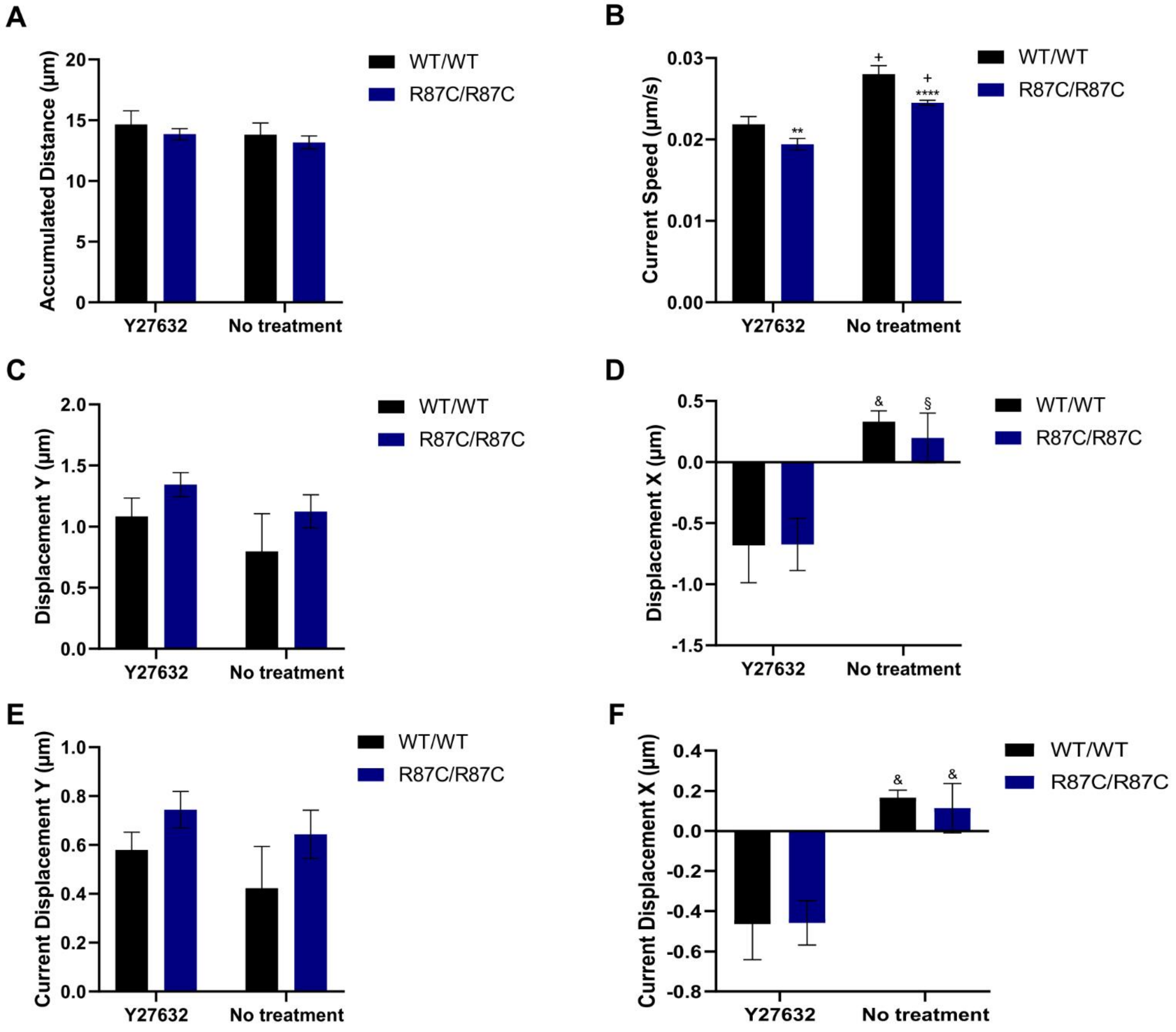


**Supplementary Fig. 5: Cell Tracking of hESC-Derived NPCs.** Analysis of (A) Accumulated Distance, (B) Current Speed, (C) Displacement Y, (D) Displacement X, (E) Current Displacement Y, and (F) Current Displacement X of hESC-derived NPCs. Statistical analysis between genotypes (WT/WT vs. R87C/R87C): **p ≤ 0.01, ****p ≤ 0.0001. Statistical analysis comparing conditions to WT/WT treated with Y-27632: § p ≤ 0.01, & p ≤ 0.001, + p ≤ 0.0001.

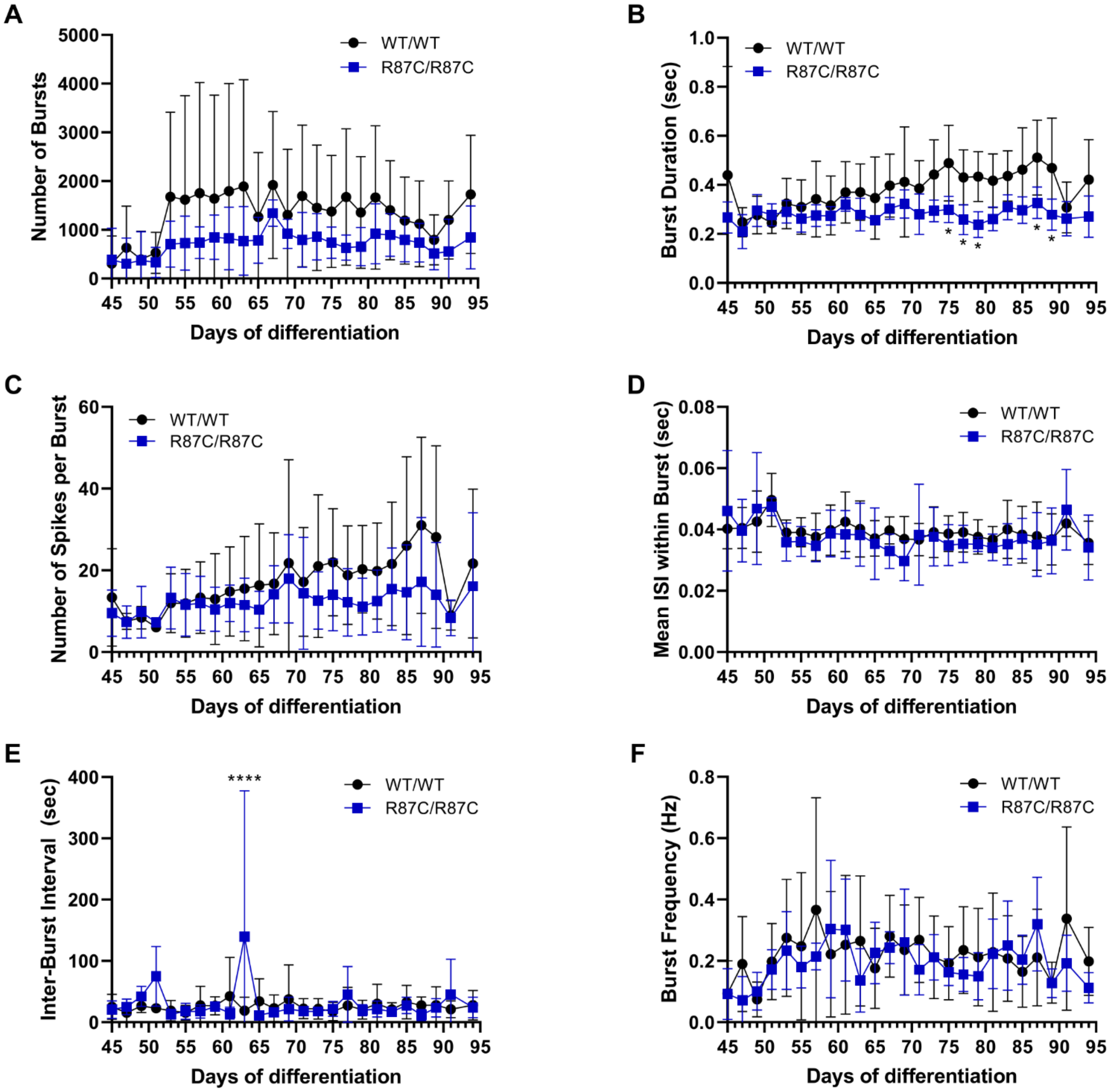


**Supplementary Fig. 6. Quantification of network burst activity in WT/WT and R87C/R87C neuronal cultures throughout differentiation.** (A) Number of Bursts—the total number of bursts detected within the recording period; (B) Burst Duration (sec)—the duration of individual bursts in seconds; (C) Number of Spikes per Burst—the number of spikes contained within each burst; (D) Mean ISI within Burst (sec)—the average inter-spike interval within a burst; (E) Inter-Burst Interval (sec)—the temporal interval between the end of one burst and the beginning of the next; (F) Burst Frequency (Hz)—the frequency of bursts per second (Hertz). Data are presented as mean ± SD. Statistical analysis was performed using two-way ANOVA with Tukey's multiple comparisons test. Significance is indicated as *p < 0.05 and ****p < 0.0001. n=4.

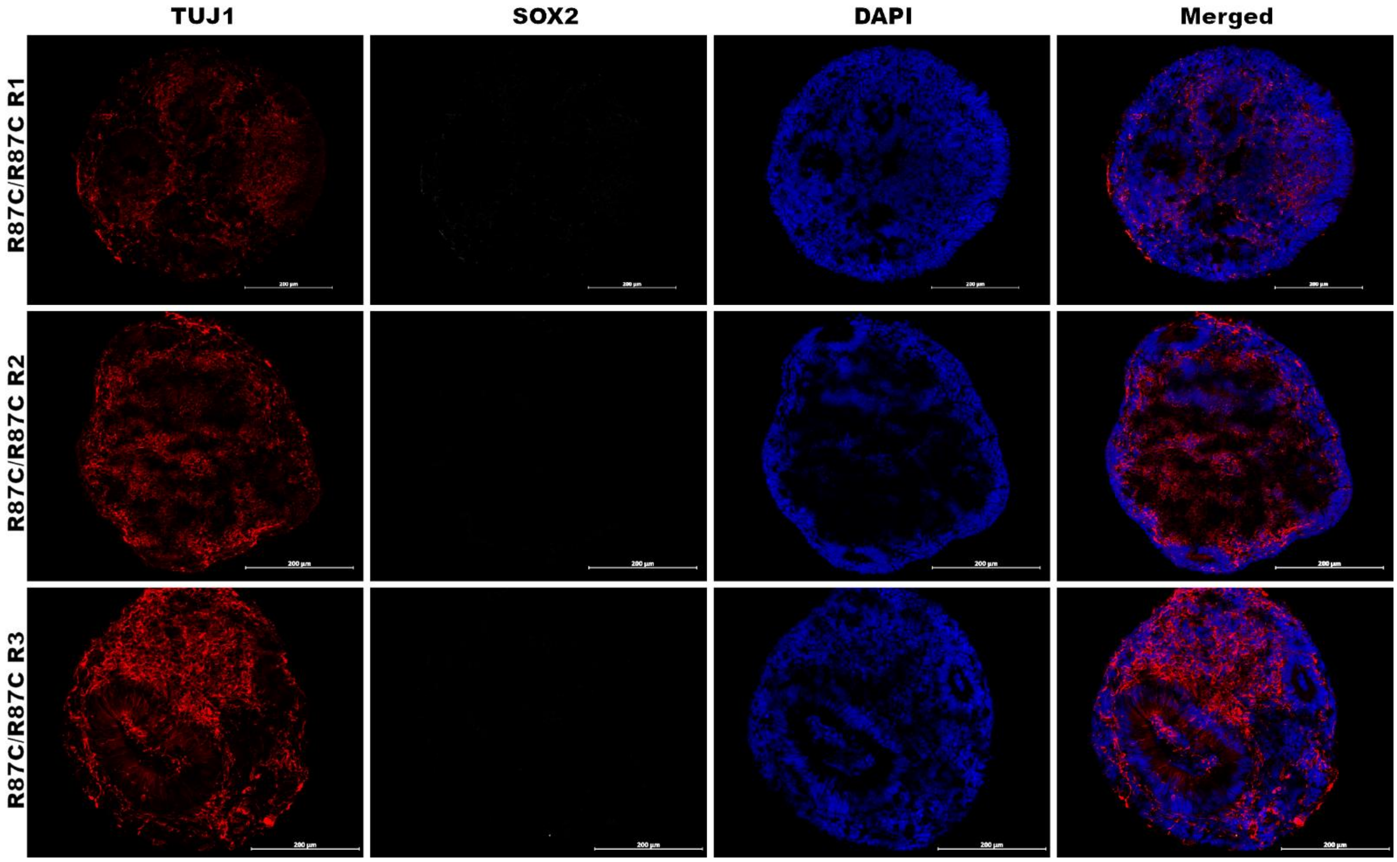


**Supplementary Fig. 7. Technical replicates of R87C/R87C organoid staining**. Representative immunofluorescence images of Day 30 cortical organoids stained for TUJ1 (red, neurons) and SOX2 (white, NPCs) across three independent replicates (R1, R2, and R3). Each replicate corresponds to an independent organoid batch. DAPI: blue. Scale bar: 200 μm.

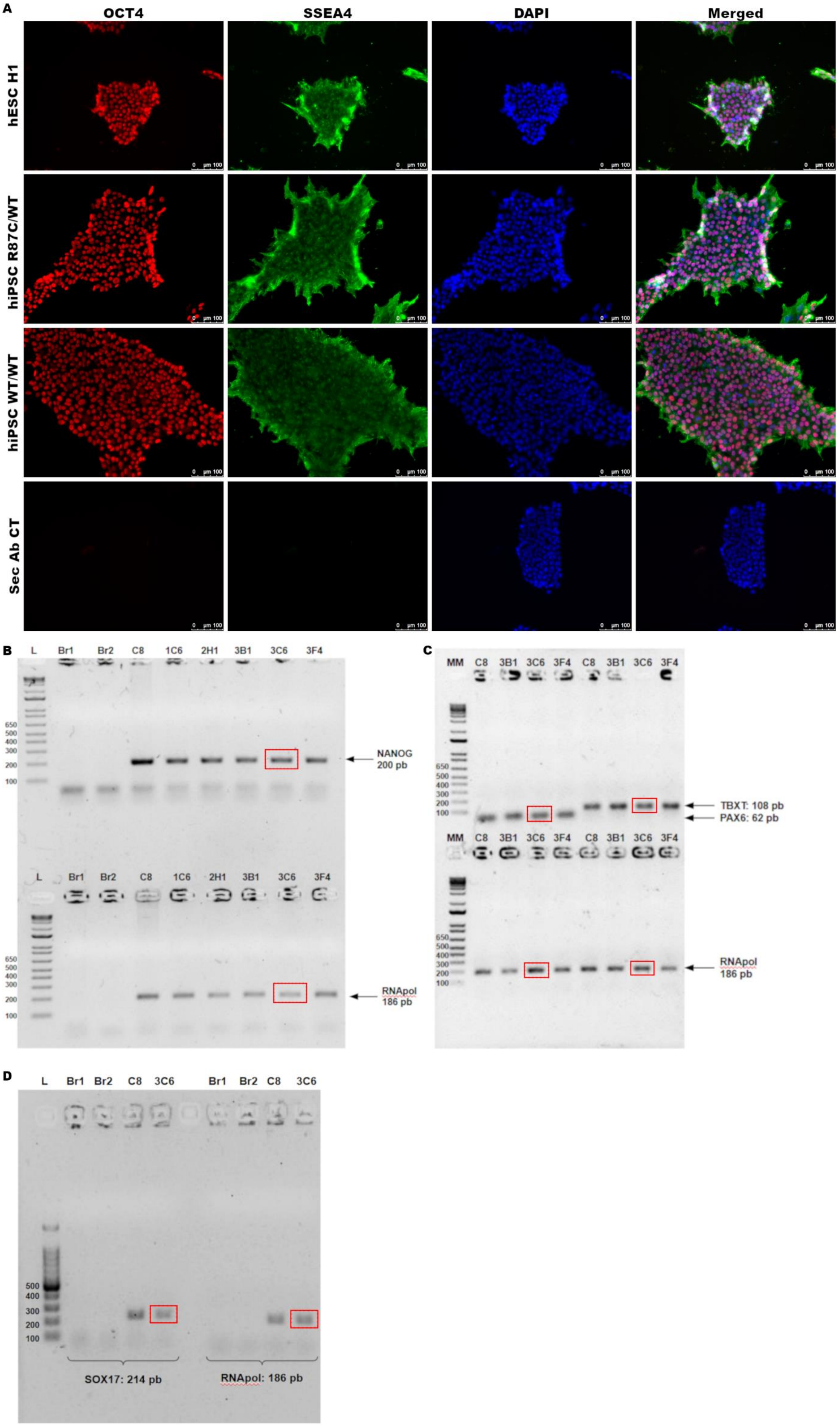
A
OCT4
SSEA4
DAPI
Merged
hESC H1
hiPSC R87C/WT
hiPSC WT/WT
Sec Ab CT
0 µm 100
B
L Br1 Br2 C8 1C6 2H1 3B1 3C6 3F4
650
500
400
300
200
100
NANOG
200 pb
RNApol
186 pb
C
MM C8 3B1 3C6 3F4 C8 3B1 3C6 3F4
TBXT: 108 pb
PAX6: 62 pb
RNApol
186 pb
D
L Br1 Br2 C8 3C6 Br1 Br2 C8 3C6
500
400
300
200
100
SOX17: 214 pb
RNApol: 186 pb

**Supplementary Fig. 8: Expanded hiPSC Characterization after CRISPR/Cas9 Gene Editing.** (A) Separate channels showing the expression of pluripotency markers in the corrected clone. The H1 cell line, a hESC, was used as a positive control. Sec Ab CT: cells incubated with only the secondary antibody as a control for nonspecific staining. (B-D) Agarose gels showing the analysis of pluripotency (B) and germ layer (C-D) markers expression in different clones after gene editing. *NANOG*: pluripotency marker*.* *PAX6:* ectoderm marker. *TBXT:* mesoderm marker. *SOX17:* endoderm marker. *GAPDH*: housekeeping gene. Ladder: 1 kb plus. The bands used in Figure 4 are outlined with a red line.

**Supplementary Data 1-4: Cell Tracking Videos (MP4 files).** Cell tracking videos of the WT hESC-derived NPCs: (1) Y-27632 treatment or (2) "No treatment" condition, and R87C hESC-derived NPCs: (3) Y-27632 treatment or (4) "No treatment" condition.
